# Hyper‐intense lesion in the pons on brain magnetic resonance imaging in a patient with diffuse large B‐cell lymphoma

**DOI:** 10.1002/jha2.251

**Published:** 2021-06-09

**Authors:** Yo Saito, Dai Maruyama, Akiko Miyagi Maeshima, Yuko Kubo, Koji Izutsu

**Affiliations:** ^1^ Department of Hematology National Cancer Center Hospital Tokyo Japan; ^2^ Department of Hematology Oncology Cancer Institute Hospital of Japanese Foundation for Cancer Research Tokyo Japan; ^3^ Department of Pathology National Cancer Center Hospital Tokyo Japan; ^4^ Department of Diagnostic Radiology National Cancer Center Hospital Tokyo Japan

A 63‐year‐old man was initially diagnosed with stage IE (paranasal cavity) diffuse large B‐cell lymphoma (DLBCL). He received three cycles of R‐CHOP therapy (rituximab, cyclophosphamide, doxorubicin, vincristine, and prednisolone) and involved field radiotherapy, followed by intrathecal methotrexate for the prophylaxis of central nervous system (CNS) involvement. After treatment, he achieved complete metabolic response (CMR) and maintained it for 8 years. At 72 years old, he presented with persistent fever and splenomegaly. Bone marrow biopsy (BMB) demonstrated the infiltration of DLBCL cells. He received six cycles of R‐CHOP therapy and achieved second CMR.

However, 1 year later, he presented with thrombocytopenia, a high LDH level, and splenomegaly. Although no infiltration of lymphoma cells was noted on BMB, random skin biopsy revealed the infiltration of DLBCL cells in the lumina of small‐sized vessels of subcutaneous fat tissue. A hyper‐intense lesion in the pons (arrow) on brain magnetic resonance imaging (MRI) was observed on T2‐weighted imaging (T2WI) (A) and diffusion‐weighted imaging (dWI) (B) (Figure 1). However, he had no neurological symptoms, and cerebrospinal fluid testing confirmed no infiltration of lymphoma cells. He received R‐GDP therapy (rituximab, gemcitabine, dexamethasone, and cisplatin). Thrombocytopenia, high LDH, and splenomegaly improved after the first cycle. Moreover, MRI demonstrated disappearance of the hyper‐intense lesion in the pons on T2WI (C) and dWI (D) after three cycles of R‐GDP therapy (Figure 1).

**FIGURE 1:**
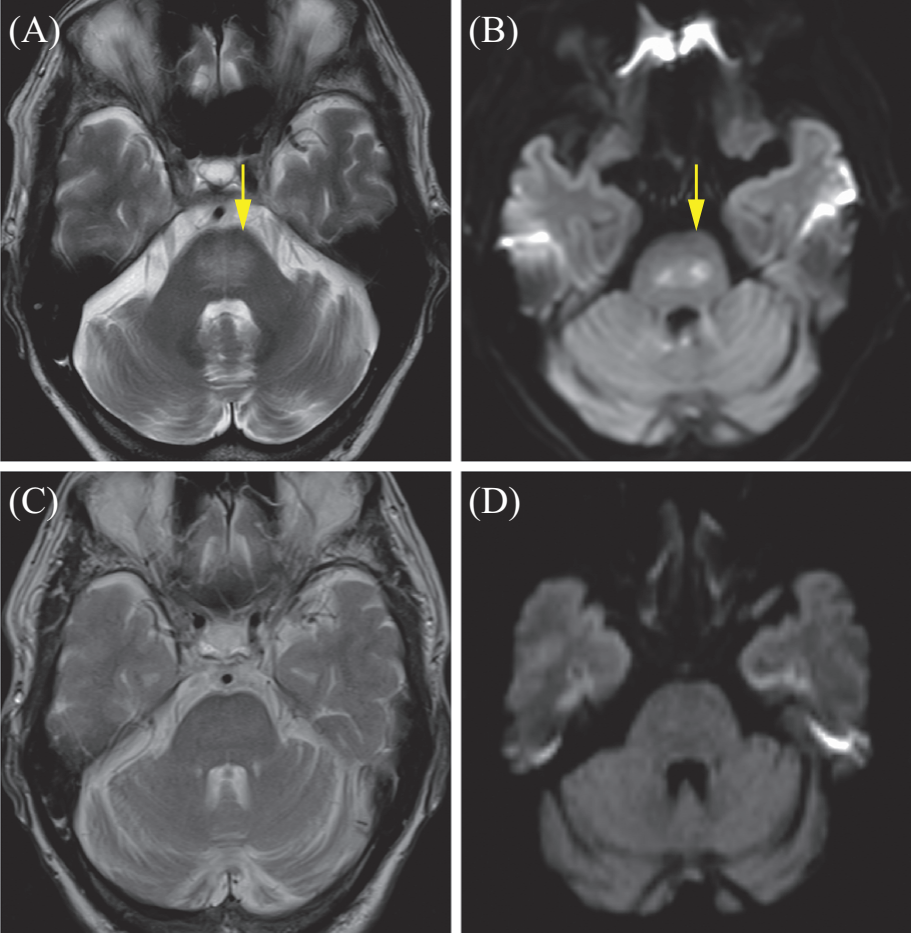
A hyper‐intense lesion in the pons (arrow) on brain magnetic resonance imaging (MRI) was observed on T2‐weighted imaging (T2WI) (A) and diffusion‐weighted imaging (dWI) (B). MRI demonstrated disappearance of the hyper‐intense lesion in the pons on T2WI (C) and dWI (D) after three cycles of R‐GDP therapy

Abe et al. reported a relationship between intravascular large B‐cell lymphoma (IVLBCL) and brain MRI findings [1]. However, the brain MRI findings of intravascular involvement by DLBCL cells are not generally known because of its rarity. At the time of the second relapse with intravascular involvement, the brain MRI findings of this patient resembled those of the patients who were previously reported by Abe et al. Our patient achieved a complete response with non‐CNS oriented chemotherapy and maintained it for more than 2 years. The hyper‐intense lesion in the pons on T2WI of MRI may have reflected the involvement and obstruction of microvessels in the pons rather than the parenchyma involvement by lymphoma cells.

## CONFLICT OF INTEREST

Dai Maruyama reports honoraria and research funding from Chugai Pharmaceutical, outside the submitted work. Yo Saito, Akiko Miyagi Maeshima, Yuko Kubo, and Koji Izutsu have no conflict of interest.

## AUTHOR CONTRIBUTIONS

Yo Saito and Dai Maruyama wrote the initial draft of the manuscript. Dai Maruyama is the attending physician of this patient. Akiko Miyagi Maeshima contributed to pathologic interpretations. Yuko Kubo contributed to radiographic image interpretations. Koji Izutsu assisted in the preparation of the manuscript. The final version of the manuscript was approved by all authors.

## Data Availability

The data that support the findings of this study are available from the corresponding author upon reasonable request.
